# The Quality of Life of Cancer Survivors: The Role of Social Factors

**DOI:** 10.3390/cancers17193145

**Published:** 2025-09-27

**Authors:** Sigríður Ása Alfonsdóttir, Harpa Lind Hjördísar Jónsdóttir, Guðfinna Halla Þorvaldsdóttir, Sigrún Elva Einarsdóttir, Jóhanna Eyrún Torfadóttir, Sigríður Gunnarsdóttir

**Affiliations:** 1 Icelandic Cancer Registry and Research Center at the Icelandic Cancer Society, 105 Reykjavík, Iceland; sigridurg@krabb.is; 2 Faculty of Psychology, School of Health Sciences, University of Iceland, 102 Reykjavik, Iceland; harpalj@hi.is; 3 The Icelandic Cancer Society, 105 Reykjavik, Iceland; halla@krabb.is (G.H.Þ.); sigrunelva@krabb.is (S.E.E.); 4 Centre of Public Health Sciences, School of Health Sciences, University of Iceland, 102 Reykjavik, Iceland; jet@hi.is; 5 Faculty of Nursing & Midwifery, School of Health Sciences, University of Iceland, 102 Reykjavik, Iceland; 6 Department of Oncology, Landspitali-National University Hospital of Iceland, 102 Reykjavik, Iceland

**Keywords:** cancer, survivorship, quality of life, determinants of health, social factors, role functioning, social functioning, social support, depression

## Abstract

Background/Objectives: With improved technology and cancer treatments, the number of cancer survivors is growing. However, many cancer survivors experience long-term effects of cancer and its treatment, leading to a high health burden and poorer quality of life (QoL). A growing body of literature has explored the relationship between sociodemographic variables and QoL among cancer patients, although findings remain mixed. This study aims to assess the impact of social factors—role functioning, social functioning and social support—on the QoL of Icelandic cancer survivors diagnosed between 2015 and 2019, stratified by gender and cancer type (breast, prostate and colorectal cancer). Method: This study is a secondary analysis study, using data from a cross-sectional study conducted by the Icelandic Cancer Society in 2020–2021. In total, 1200 cancer survivors participated. Results: Multiple regression showed that less depressive symptoms, higher role and social functioning, better instrumental social support, higher educational attainment and income, less financial difficulties and lower age were significantly associated with higher QoL among participants. Conclusions: cancer survivors’ global health status/QoL is highly influenced by role functioning and depression symptoms, while other predictive factors, including social functioning, social support, education and income, vary by gender and cancer type.

## Simple Summary

In 2022, roughly 54 million people worldwide were alive within 5 years of receiving a cancer diagnosis and, with improved technology and treatments, this group is growing fast. However, cancer survivors often experience higher health burden and poorer quality of life (QoL) compared to the general population. Little is known about what contributes to these poor health outcomes, especially what role the social factors might play. This study aims to assess the impact of social factors—including role functioning, social functioning and social support—on the QoL of cancer survivors. This secondary analysis of a cross-sectional study called “The Compass” by the Icelandic Cancer Society included 1200 cancer survivors. The analyses were stratified by gender and cancer type (breast, prostate and colorectal). Taking gender and cancer type-based predictors into consideration in survivorship care could improve patients’ health-related quality of life.

## 1. Introduction

As of 2022, roughly 54 million people worldwide were alive within 5 years of receiving a cancer diagnosis [1]. Additionally, 20 million people received a new diagnosis and 9.7 million people died [2]. The most commonly diagnosed cancer types are breast, lung, colorectal and prostate [3]. In Iceland, between 2020 and 2024, an average of 2055 people were diagnosed with cancer annually, with incidence rates of 615 per 100,000 for men and 552 per 100,000 for women. The mortality rates were 218.7 per 100,000 for men and 182.6 per 100,000 for women, while the five-year overall survival rate reached 70% [4].

With the aging population and advances in cancer detection, technology and treatments, the number of cancer survivors is growing [5,6]. However, cancer survivors often experience an array of challenges that affect their overall health and well-being, including physical [7,8], emotional [8,9,10,11,12] and social dimensions [12,13,14,15,16,17]. As the number of survivors continues to increase, attention is shifting to long-term quality of life (QoL).

Quality of life is a multidimensional concept that encompasses all aspects of a person’s well-being. A closely related concept, health-related quality of life (HRQoL), is a more specific subset that specifically refers to individuals’ perceptions of their physical, emotional, mental and functional well-being, and how these affect their daily life, including work, relationships, and leisure [18]. Two core components of HRQoL are role and social functioning. Social functioning refers to an individual’s ability to interact with their social environment and fulfill their social roles [19]. Role functioning refers to how well an individual performs in the various roles they occupy in their life, such as those related to family, workplace, and community. Limited research has specifically addressed the role of these two factors in cancer survivorship, but studies show that impairments in these areas can significantly affect overall well-being and life satisfaction [20]. While increasing survival rates is the primary goal of treatment, an equally important objective is maintaining quality of life during and after cancer treatment and preventing its deterioration.

A growing body of literature has explored the relationship between sociodemographic variables and QoL among cancer patients, although the findings remain mixed. Some studies have found a significant relationship between QoL and age [21,22,23,24,25,26], gender [22,25,26,27,28,29,30,31,32,33], education [21,25,34,35,36], economic condition [21,25], marital status [21], employment [26,28] and family size [37], while others have found no such association [25,38,39,40,41]. Despite this, gender differences seem to play a critical role in shaping the QoL and social experiences of cancer survivors. Studies examining QoL predictors reveal both gender-specific predictors and common predictors. The QoL of female cancer survivors is predicted by pain intensity [42,43], body image concerns [43], financial strain, employment status and optimism [44], while male survivors’ QoL is more strongly predicted by limited health literacy [45] and sexual health issues [43]. Common predictors of QoL include cognitive impairment [45], energy level, social support and depression [42,44,45]. Depression has consistently been shown to negatively affect QoL in cancer patients. High rates of depression have been reported in this population [8,9], and depression has been associated with poorer treatment outcomes and increased cancer-specific and all-cause mortality [10,11,46,47].

Men and women tend to adopt different coping mechanisms, often reflecting existing gender norms [48]. While men tend to favor problem-solving or avoidance [49,50,51], women favor emotional coping strategies, such as sharing experiences and seeking social support [48,50,51,52,53,54]. These gendered patterns may affect how individuals respond to illness, express their experiences and seek help [55]. From a public health perspective, understanding these gendered patterns is essential for developing tailored interventions.

Social factors play a crucial role and have been termed one of the most important factors in determining the QoL in chronic illness [12,44]. A cancer diagnosis can disrupt social functioning, reduce social participation, and alter relationships. Survivors often experience decreased energy and motivation for social activities, a diminished social network, and changes in social roles [56]. However, strong social ties and support networks have been shown to enhance QoL, improve health outcomes, reduce mortality risk [14,15,16,17], increase life satisfaction and lower levels of depression [15,57]. Survivors with higher QoL tend to experience greater social and role functioning [29,37,58,59,60,61,62,63,64,65] and lower levels of social isolation [15,66] compared to those with lower QoL.

Social support is a key factor when adapting to a cancer diagnosis [35]. Research on social support and health show that experiencing high levels of social support is associated with a 25% decrease in relative risk for cancer mortality [17] and higher QoL [15,27,29,35]. Social support has four subtypes, all of which are predictive of overall QoL, including emotional and instrumental social support [67]. Emotional social support addresses a person’s emotional and psychological needs, expressing care, concern, empathy and understanding, while instrumental social support involves providing practical assistance to someone to manage their immediate needs. This includes offering a ride to a doctor’s appointment and helping with chores [68]. These two types of social support are associated with higher social and emotional well-being [15].

Study Aim

Given the increasing numbers of cancer survivors, it is essential to identify the social factors that contribute to better quality of life. This study aims to explore the association between social factors and QoL among cancer survivors in Iceland, with a specific focus on gender and the three most common cancer types in the sample (breast cancer, prostate cancer and colorectal cancer). The analyses will be stratified by gender and cancer type to explore the association between social factors and QoL in more detail and to identify potential areas for intervention to improve QoL in this growing population.

## 2. Materials and Methods

### 2.1. Participants

This is a secondary analysis of existing data, utilizing data from the “Compass” (“Áttavitinn”), a cross-sectional study conducted by the Icelandic Cancer Society. In June, all individuals (18–80+ years old) who had been diagnosed with cancer between 2015 and 2019 were invited to participate in this study via a mailed invitation letter. Information on cancer participants was gathered from the Cancer Registry of Iceland, and participation consisted of answering a questionnaire, either electronically or in print, with informed consent obtained. The data was collected between 8 June 2020 and 1 May 2021. A total of 4575 individuals received an invitation to participate in this study, and a total of 1840 individuals answered the questionnaire, yielding a 40% response rate [69].

**Figure 1:**
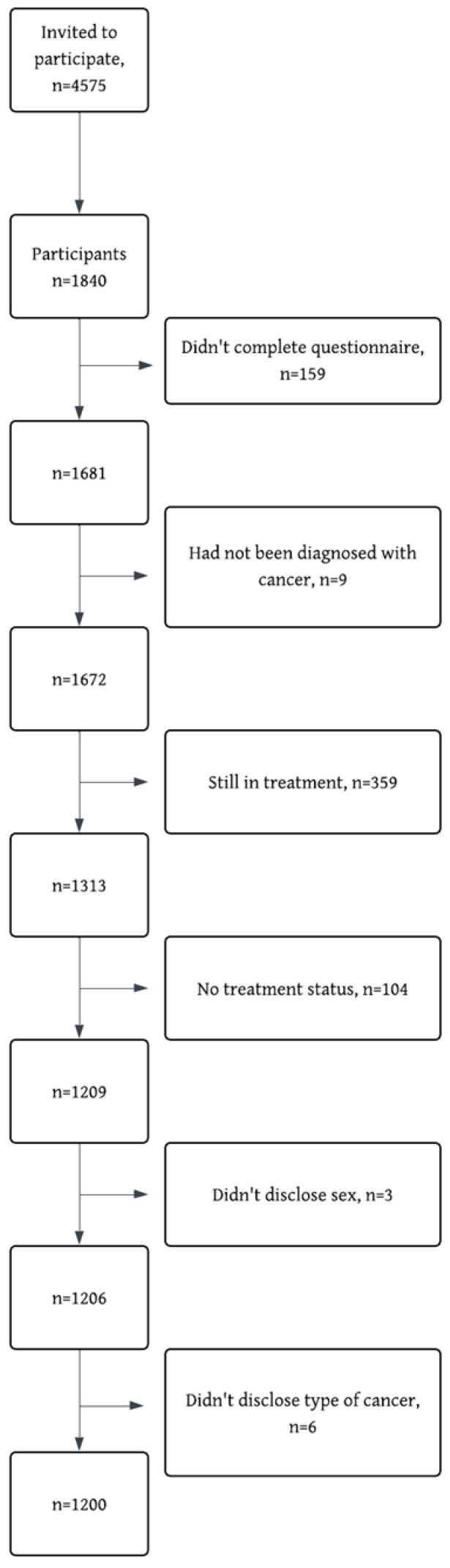
STROBE flow diagram of reasons for exclusion and missing data.

For the present study, only individuals who had completed their cancer treatment were included, *n* = 1200 (Figure 1). To conduct an analysis based on cancer type, the three most common cancer types were selected, including breast cancer (BC, *n* = 391), prostate cancer (PC, *n* = 167) and colorectal cancer (CC, *n* = 147).

### 2.2. Instruments

#### 2.2.1. Demographic Characteristics

Information about participants‘ age and gender was gathered from Registry Iceland, while level of education, marital status, number of children, occupational status, income and number of treatments were obtained via the questionnaire.

#### 2.2.2. EORTC-QLQ-C30

The EORTC-QLQ-C30 is a widely used 30-item questionnaire that assesses the health-related QoL of cancer patients [70] and includes items measuring QoL (termed “global health status/QoL” in the questionnaire), role functioning, social functioning and financial difficulties. The questionnaire has adequate reliability, with a Cronbach’s alpha of 0.56 to 0.90 in previous studies [71,72,73] and 0.91 in the current study. The alpha for individual scales was adequate, with 0.90 for the global health status/QoL scale, 0.91 for role functioning and 0.89 for social functioning.

The 30 items are divided into five functional scales, nine symptom scales and one global health status/QoL scale. In addition, a QLQ summary score can be calculated based on all items. However, it is not advised to use this score for further analysis, but rather use the global health status/QoL scale as the outcome variable [74], as is applied in this study. Each scale is measured by between one and five items, with the responses rated on a 4-point Likert scale, ranging from 1 = Not at All to 4 = Very Much. The only exception to this is the global health status/QoL scale, which is rated on a 7-point Likert scale ranging from 1 = Very poor to 7 = Excellent. Scores were converted to a 0–100 scale according to the EORTC scoring manual, where higher scores indicate better quality of life or functioning. Missing values were imputed using the mean of the completed items within a scale, provided that at least half of the items in that scale had been answered, in line with EORTC recommendations [74]. Previous research has established thresholds for clinical importance (TCIs) that vary across the questionnaire’s subscales. For example, scores below 58 on role or social functioning indicate clinically important impairment, while scores above 17 on financial difficulties reflect a clinically important problem [75]. When evaluating change over time, a 10-point difference has often been used as a threshold for meaningful change. However, this cut-off has been debated, and consensus on the interpretation of QLQ-C30 scores is still lacking [76,77].

#### 2.2.3. PHQ-9

Patient Health Questionnaire (PHQ-9) measures symptoms of depression and contains 9 items intended to screen for and measure depression severity [78]. The response scale is coded as 0 = Not at all, 1 = Several days, 2 = More than half the days, 3 = Nearly every day. A total score is calculated by summing responses. A score of 1–4 indicates minimal depression; 5–9 mild depression; 10–14 moderate depression; 15–19 moderately severe depression; and 20–27 severe depression [78]. In this study, the total score was used in the analysis to represent symptoms of depression. The questionnaire has adequate reliability, with a Cronbach’s alpha of 0.86 to 0.89 in previous studies [78,79] and 0.90 in the current study.

#### 2.2.4. The Compass

The Compass is a 110-item survey tool based on a Danish survey called “the Barometer survey” [80] and has been adapted to Icelandic society. The survey comprises questions assessing demographic factors and experiences with the diagnostic process and cancer treatment. In this study, information on the level of education, marital status, number of children, occupational status, personal income and number of treatments obtained via the questionnaire was used. Additionally, variables measuring emotional and instrumental social support were used (Table 1).

### 2.3. Statistical Analysis

Statistical analyses were performed in R studio, version 2025.05.0. Descriptive statistics for the sample characteristics, outcome variable and covariates were calculated (Table 2), followed by a correlation analysis (Table 3). To score the variables from the EORTC-QLQ-C30 scale, global health status/QoL and role and social functioning, a package called PROscorer was used. The package is specifically designed to score patient-reported outcomes such as the QLQ-C30 questionnaire. In the case of missing data, mean imputation was used and performed by the PROscorer package, as directed by the EORTC manual.

To explore the association between social variables and the global health status/QoL of cancer survivors following cancer therapy, a multiple linear regression was conducted using the enter method. Social factors (role and social functioning, instrumental and emotional social support) and depression were entered into the model simultaneously to assess their individual contributions to the outcome, while controlling for the effects of the other variables (demographic) in the model. Standardized beta coefficients were reported to indicate the strength and direction of the associations between the predictors and the outcome. The analysis was stratified by gender for the whole sample to explore gender differences and by cancer type to explore cancer-specific relationships.

This study had adequate power to conduct the proposed analyses across the three major cancer groups. However, after more in-depth analyses, the PC and CC groups had decreased due to missing data in the social support variable (PC, n = 28 and CC, n = 28). Therefore, ad hoc power analysis was performed in Gpower,(version 3.1.9.6) which confirmed that this study still had adequate power. To improve power and to increase the sample size for those two groups, we removed the variable with the highest number of missing data (*instrumental social support*) from the analysis for these two groups but included it in the analysis for the breast cancer group.

## 3. Results

### 3.1. Socio-Demographic Characteristics

Roughly 60% of the sample were female, the average age was 62.0 years (SD = 11.8, range 23–85), and the mean age at diagnosis was 58.9 years old (SD = 11.9, range 18–80). Most of the sample lived in the greater Reykjavík area (64.1%) and were married or cohabiting (79.2%). More than a third of the sample had a university degree (38.9%) and this trend was consistent across all three cancer types. Compared to the other cancer types, the individuals in the breast cancer group were, on average younger, diagnosed earlier, more likely to have children under the age of 18 at home, had undergone more treatment types and experienced greater financial difficulties (Table 2).

The average score on the global health status/QoL scale was 72.3 among all cancer types, 70.4 among women and 75.1 for men, all of which are higher than published reference values (61.3) [81]. There was a significant difference between global health status/QoL scores among the three cancer types, with 70.6 points among breast cancer survivors, 77.0 among prostate cancer survivors and 73.4 among colorectal cancer survivors. On average, the prostate cancer survivors had the highest average score on global health status/QoL, role functioning, social functioning and emotional social support, compared to the other two cancer types (Table 2).

Regarding depression symptoms, a total of 14.7% of breast cancer survivors experienced moderate-to-severe depression symptoms, compared to 6.9% among the colorectal cancer survivors and 3.7% of the prostate cancer survivors. The majority of the participants had minimal symptoms of depression (65%).

### 3.2. Inferential Statistics

**Figure 2:**
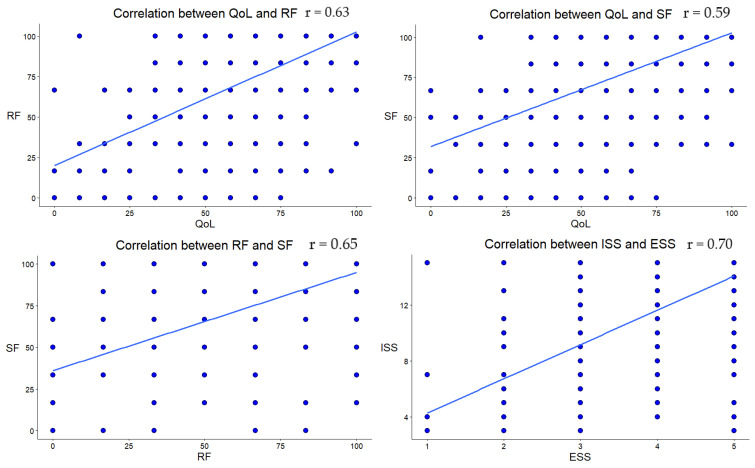
Scatterplot of the highest significant correlation coefficients. The blue dots represent individual scores on each variable.

To analyze the association of social variables with global health status/QoL, a correlation matrix using Pearson correlation was generated for all variables of interest (Table 3). Most correlations were small (|r| < 0.30), although a higher correlation was observed between some variables: SF and RF (r = 0.65, *p* < 0.001), QL and SF (r = 0.59, *p* < 0.001), QL and RF (r = 0.63, *p* < 0.001) and ISS and ESS (r = 0.70, *p* < 0.001) (Figure 2). Despite several correlations reaching statistical significance, the effect sizes indicated weak associations overall. The correlations among the predictors were well below levels of concern for multicollinearity (r < 0.80), and therefore all variables were retained for subsequent analyses.

#### 3.2.1. All Survivors

**Figure 3:**
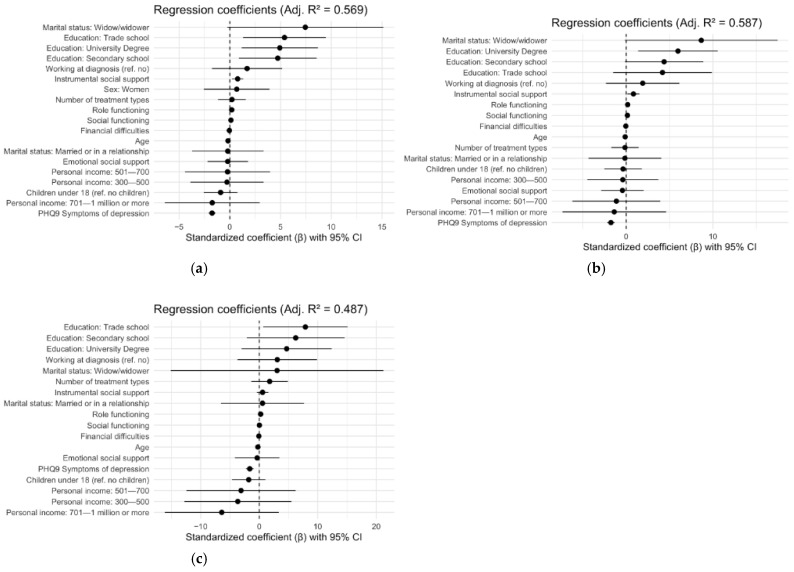
Forest plots illustrate the results from the regression analysis from Table 4. The following panels show (**a**) regression analysis, group: all survivors; (**b**) regression analysis, group: women; (**c**) regression analysis, group: men.

The results of multiple regression models for global health status/QoL showed that age, education, financial difficulties, role and social functioning, instrumental social support and depression were all significant predictors of global health status/QoL among all cancer survivors (Table 4 and Figure 3). The analysis shows that older age (β = −0.11, *p* = 0.01), experiencing more financial difficulties (β = −0.08, *p* = 0.02) and more depression symptoms (β = −0.42, *p* ≤ 0.001) were negatively associated with global health status/QoL, while better role functioning (β = 0.26, *p* ≤ 0.001), higher education levels (secondary school = β = 0.23, *p* = 0.02; trade school education = β = 0.26, *p* = 0.01; university degree = β = 0.24, *p* = 0.01), better social functioning (β = 0.14, *p* ≤ 0.001) and better instrumental social support (β = 0.12, *p* = 0.01) were all positively associated with global health status/QoL.

#### 3.2.2. Gender Differences

Among women, global health status/QoL was most negatively affected by experiencing depressive symptoms (β = −0.41, *p* ≤ 0.001), while having university education (β = 0.28, *p* = 0.01), better role functioning (β = 0.23, *p* ≤ 0.001), better social functioning (β = 0.18, *p* ≤ 0.001) and receiving more instrumental social support (β = 0.12, *p* = 0.03) were positively associated with global health status/QoL.

Among men, having trade school education (β = 0.41, *p* = 0.02) and better role functioning (β = 0.35, *p* ≤ 0.001) were positively associated with global health status/QoL, while experiencing depressive symptoms was negatively associated, β = −0.39, *p* ≤ 0.001.

Educational level, role functioning and depression predicted global health status/QoL among both men and women, while social functioning and instrumental social support additionally predicted women’s global health status/QoL (Table 4 and Figure 3).

#### 3.2.3. Cancer Type

**Figure 4:**
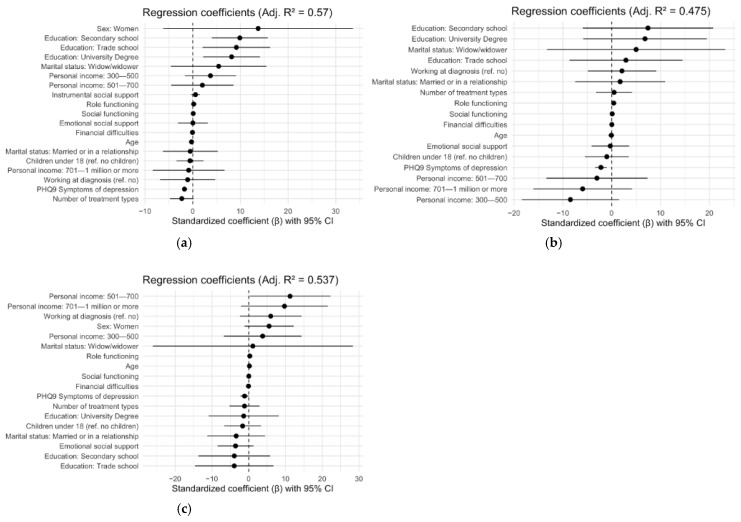
The forest plots illustrate the results from the regression analysis from Table 5. The following panels show (**a**) regression analysis, group: breast cancer survivors; (**b**) regression analysis, group: prostate cancer survivors; (**c**) regression analysis, group: colorectal cancer survivors.

Role functioning and depression were significant predictors of global health status/QoL across all cancer types of interest. However, the demographic predictors associated with global health status/QoL varied by cancer type (Table 5 and Figure 4). The multiple regression analysis indicated that a better global health status/QoL among breast cancer survivors was positively associated with higher educational levels (secondary school, β = 0.50, *p* = 0.001; trade school education, β = 0.46, *p* = 0.012; university degree, β = 0.41, *p* = 0.008) and better role functioning, (β = 0.27, *p* ≤ 0.001), while experiencing more depressive symptoms was negatively associated with global health status/QoL, β = −0.41, *p* ≤ 0.00. For prostate cancer survivors, better role functioning was positively associated with global health status/QoL (β = 0.47, *p* ≤ 0.001), while experiencing more symptoms of depression was negatively associated with global health status/QoL scores (β = −0.37, *p* ≤ 0.001). Among colorectal cancer survivors, having a medium income was positively associated with global health status/QoL (β = 0.63, *p* = 0.045), along with better role functioning (β = 0.47, *p* ≤ 0.001), while experiencing more symptoms of depression was negatively associated with scores (β = −0.23, *p* = 0.046) (Table 5 and Figure 4).

## 4. Discussion

The average global health burden/QoL score of the sample was 72.3 points on a scale from 0 to 100, which is above existing reference values. The scores differed significantly between cancer types and gender and were predicted by different demographic and social factors. Across the entire sample, the strongest predictor of global health status/QoL was the presence of depressive symptoms, followed by educational attainment, role functioning, social functioning, instrumental social support, age and financial difficulties. These results are consistent with previous research, highlighting the detrimental impact and high prevalence of depression on QoL [10,11,46,47], the positive associations between enhanced role and social functioning and higher QoL [29,37,58,59,60,61,62,63,64,65] and between greater social support and improved QoL [15,27,29,35].

Regarding socioeconomic variables—specifically education, income and financial difficulties—our findings indicate that individuals with better socioeconomic conditions generally report higher levels of QoL. In particular, higher educational attainment and fewer financial difficulties were associated with better QoL outcomes across the sample. These results are consistent with previous studies [82,83].

### 4.1. Gender Differences

An important contribution of the present study is the identification of gender differences in social predictors of global health status/QoL. The global health status/QoL of both men and women is highly associated with both depression, role functioning and education. Among men, role functioning was the only significant social predictor, whereas for women, role functioning, social functioning and instrumental social support were all significant predictors. These findings suggest that women’s global health status/QoL may depend more on the quality and availability of social networks and support systems compared to men. This is consistent with previous research on gender differences in coping styles, which emphasizes women’s greater reliance on social support and interpersonal sharing [5,48,50,51,52,53,54]. Such differences may help explain the broader influence of social variables observed among female survivors.

### 4.2. Cancer Specific Predictors

Global health status/QoL among all cancer types was highly associated with depression and role functioning. However, this study identified cancer-specific differences in demographic predictors. Global health status/QoL was linked to educational level in breast cancer survivors but not in prostate or colorectal cancer survivors. Among the colorectal survivors, having a medium personal income was associated with higher global health status/QoL compared with the lowest personal income group. This association was not observed in other cancer groups. This indicates that the influence of demographic factors on global health status/QoL is not universal but may instead reflect challenges specific to certain cancer diagnoses, populations and treatments. Considering these diagnosis-specific factors in survivorship could help improve outcomes.

### 4.3. Importance of Social Variables

The consistent predictive value of role functioning across cancer types underscores the critical role that returning to everyday activities plays in enhancing QoL after surviving cancer. Role functioning represents an essential dimension of quality of life, as it connects individuals to purpose, identity and social contribution. In addition to fulfilling basic tasks, role functioning reflects a sense of purpose in life, such as being a parent to one’s children, being a useful worker or member of society, and the broader experience of having a valued role in society. These components are not only integral to individual identity and self-worth but also to social relationships, highlighting the importance of role functioning as a key factor in both clinical and public health contexts. Disruptions in role functioning can therefore have wide-ranging implications for both individual adjustment and collective life [84]. Interventions to restore role functioning include targeted peer groups, patient organizations that facilitate reintegration into daily life, and structured exercise programs [85]. However, the success of these efforts often depends on addressing underlying psychological challenges.

This emphasizes the need for comprehensive strategies that combine targeted rehabilitation, support groups and mental health interventions to help survivors re-engage with daily roles despite potential limitations. In particular, the strong influence of depressive symptoms on global health status/QoL across cancer groups underscores the importance of systematic mental health screening and support. Early identification and treatment of depressive symptoms may improve overall outcomes for cancer survivors.

### 4.4. Strengths and Limitations

This study’s strengths include a relatively large sample, which enabled testing of a comprehensive model incorporating both psychosocial and demographic variables, as well as a diverse sample including multiple cancer types.

However, some limitations should be acknowledged. A key limitation is the uncertainty regarding how representative the study sample is of the entire population of cancer patients in Iceland, given that over a third of the participants had a university degree, potentially introducing response bias. Participation required computer literacy and access to electronic IDs, which may have introduced selection bias [86]. Despite the rather large sample, specific cancer groups had relatively small sample sizes, which may have limited the ability to examine certain variables. The cross-sectional design prevents conclusions about causality, and the reliance on self-reported data may have introduced reporting bias. Furthermore, in relation to gender-specific analysis, it would have been more relevant to evaluate a cohort of cancer survivors of both sexes treated for the same anatomical location (e.g., lung cancer), rather than for gender-specific cancers such as breast and prostate. However, this was not feasible due to small sample sizes. Such an approach would have allowed a more direct comparison of factors influencing quality of life by reducing bias related to cancer type, treatment regimen and long-term side effects. Finally, although multiple cancer types were included in the analysis, this study did not account for type of treatment or time since diagnosis, both of which could influence global health status/QoL.

Future research should continue to investigate the impact of social factors on the QoL of cancer survivors by gender, time since diagnosis and various cancer and treatment types in longitudinal studies to better understand the association between social predictors and QoL.

## 5. Conclusions

In conclusion, cancer survivors’ global health status/QoL is influenced by a range of social, demographic and psychological factors, underscoring the importance of a comprehensive approach to survivorship care that is tailored to cancer type and gender. Role functioning is a central dimension of quality of life, as it anchors purpose, identity and social contribution. It reflects the capacity to parent, to contribute productively and to occupy valued roles within society—all of which are vital elements of self-worth and social connectedness. Disruptions in role functioning can therefore undermine both individual well-being and collective adjustment. Considering the predictive factors identified in this study, women may particularly benefit from programs that enhance role and social functioning and improve access to instrumental support, while men may benefit more from interventions focused primarily on role functioning. Interventions aimed at restoring role functioning and addressing depression are likely to benefit all survivors, regardless of gender or cancer type.

## Figures and Tables

**Table 1:** Variable description.

Variable	Questions	Response Scale
Emotional social support	“How much or little can you trust your close family, friends or acquaintances to discuss your disease or your well-being”,	1 = Not at all, 2 = A little, 3 = To some extent, 4 = For the most part, 5 = Fully
Instrumental social support	“How much or little can you trust your close family, friends or acquaintances…” - “To assist you with things such as chores and grocery shopping” - “To assist you with matters regarding your disease and treatment, i.e., to get to a doctor and assist with taking correct medication?”, - “To assist with legal matters?”	For each question: 1 = Not at all, 2 = A little, 3 = To some extent, 4 = For the most part, 5 = Fully The summary score was a sum of scores for the three questions, 1–15

**Table 2:** Descriptive statistics for participants who finished cancer therapy.

Variables	Range	All Cancer Types	Breast Cancer	Prostate Cancer	Colorectal Cancer		All Other Cancer Types
		*n* = 1200	*n* = 391 (33%)	*n* = 167 (14%)	*n* = 147 (12%)	*p* ^a^	*n* = 495 (41%)
Gender, n (%)						<0.001	
Men		485 (40.4)	4 (1.0)	167 (100)	91 (61.9)		223 (45.1)
Women		715 (59.6)	387 (99.0)	0 (0.0)	56 (38.1)		272 (54.9)
Age, M (SD)	23–85	62.0 (11.8)	60.6 (10.7)	69.1 (7.0)	65.4 (9.3)	<0.001	59.8 (13.3)
Age at diagnosis, M (SD)	18–80	58.9 (11.9)	57.5 (10.5)	65.9 (7.0)	62.5 (9.1)	<0.001	56.6 (13.6)
Education, n (%)						<0.001	
Primary education		211 (19.1)	78 (21.3)	14 (9.0)	21 (15.9)		98 (21.6)
Secondary school		227 (20.5)	89 (24.3)	19 (12.3)	34 (25.8)		85 (18.7)
Trade school		238 (21.5)	46 (12.6)	73 (47.1)	26 (19.7)		93 (20.5)
University degree		431 (38.9)	153 (41.8)	49 (31.6)	51 (38.6)		178 (39.2)
Marital status, n (%)						0.004	
Married or in a relationship		934 (79.2)	286 (74.7)	143 (87.7)	118 (81.4)		387 (79.3)
Single		183 (15.5)	65 (17.0)	16 (9.8)	22 (15.2)		80 (16.3)
Widowed		62 (5.3)	32 (8.4)	4 (2.5)	5 (3.4)		21 (4.3)
Children under 18, n (%)						0.005	
One or two children		160 (13.5)	55 (14.3)	7 (4.2)	14 (9.6)		84 (17.1)
Three or more children		47 (4.0)	17 (4.5)	5 (3.0)	2 (1.4)		23 (4.7)
Residence, n (%)						0.534	
Capital area		760 (64.1)	239 (62.1)	100 (60.6)	100 (68.5)		321 (65.5)
Urban area (<5.000)		312 (26.3)	104 (27.0)	52 (31.5)	35 (24.0)		121 (24.7)
Urban area (>5.000)		54 (4.6)	18 (4.7)	7 (4.2)	6 (4.1)		23 (4.7)
Rural area (<200)		60 (5.1)	24 (6.2)	6 (3.6)	5 (3.4)		25 (5.1)
Personal income (ISK), n (%)						<0.001	
0–300 thousand		232 (21.7)	94 (26.7)	23 (14.7)	21 (16.9)		94 (21.4)
301–500 thousand		346 (32.3)	131 (37.2)	38 (24.4)	35 (28.2)		142 (32.3)
501–700 thousand		251 (23.4)	78 (22.2)	35 (22.4)	27 (21.8)		111 (25.3)
701–1 million or more		242 (22.6)	49 (13.9)	60 (38.5)	41 (33.0)		92 (21.0)
Working at diagnosis, n (%)		817 (68.8)	270 (70.1)	108 (65.9)	96 (65.3)	0.412	343 (69.9)
Number of treatment types, n (%)						<0.001	
M (SD)		2.06 (1.02)	2.9 (0.9)	1.5 (0.8)	1.7 (0.7)		1.65 (0.8)
No treatment		9 (0.8)	2 (0.5)	0 (0.0)	1 (0.7)		6 (1.2)
One treatment type		425 (35.4)	19 (4.9)	101 (60.5)	64 (43.5)		241 (48.7)
Two treatment types		384 (32.0)	98 (25.1)	47 (28.1)	60 (40.8)		179 (36.2)
Three or more treatment types		382 (31.8)	272 (69.5)	19 (11.4)	22 (15.0)		69 (13.9)
Quality of life, M (SD)							
EORTC Global Health Status/QoL	0–100	72.3 (20.6)	70.6 (20.4)	77.0 (18.1)	73.4 (20.5)	0.006	71.8 (21.4)
Social variables, M (SD)							
EORTC Role Functioning	0–100	79.5 (27.3)	76.6 (26.9)	87.5 (21.7)	78.5 (29.1)	<0.001	79.5 (28.2)
EORTC Social Functioning	0–100	83.0 (24.8)	81.8 (24.9)	87.2 (21.8)	82.6 (24.8)	0.094	82.8 (25.6)
EORTC Financial Difficulties	0–100	30.9 (32.8)	34.3 (32.0)	20.5 (28.9)	30.0 (32.6)	<0.001	32.0 (34.1)
Emotional social support	0–5	4.5 (0.9)	4.4 (0.9)	4.7 (0.7)	4.6 (0.7)	0.002	4.4 (0.9)
Instrumental social support	0–15	12.8 (3.1)	12.5 (3.2)	13.5 (3.1)	13.6 (2.3)	0.063	12.7 (3.2)
PHQ-9 symptoms of depression, n (%)						<0.001	
Minimal		758 (65.0)	224 (59.1)	128 (79.0	107 (73.8)		298 (62.6)
Mild		272 (23.4)	100 (26.2)	28 (17.3)	28 (19.3)		116 (24.4)
Moderate		86 (7.4)	32 (8.7)	6 (3.7)	6 (4.1)		39 (8.2)
Moderately severe		37 (3.2)	19 (5.0)	0 (0.0)	3 (2.1)		16 (3.4)
Severe		12 (1.0)	4 (1.0)	0 (0.0)	1 (0.7)		7 (1.5)

^a^ Difference between the three cancer types.

**Table 3:** Correlation matrix for the variables in the regression model.

	Age	NrT	Edu	PI	W	FI	RF	SF	ISS	ESS
Age	-									
	-									
NrT	−0.16									
	<0.001									
Edu	−0.15	−0.00								
	<0.001	0.987								
PI	−0.12	−0.06	0.41							
	<0.001	0.07	<0.001							
W	−0.42	0.11	0.16	0.40						
	<0.001	<0.001	<0.001	<0.001						
FI	−0.19	0.23	−0.07	−0.16	0.06					
	<0.001	<0.001	0.04	<0.001	0.06					
RF	0.02	−0.16	0.12	0.17	0.10	−0.37				
	0.46	<0.001	<0.001	<0.001	<0.001	<0.001				
SF	0.13	−0.20	0.02	0.06	0.02	−0.41	0.65			
	<0.001	<0.001	0.50	0.05	0.44	<0.001	<0.001			
ISS	0.12	−0.11	−0.07	0.04	−0.04	−0.28	0.17	0.23		
	0.003	0.003	0.08	0.29	0.33	<0.001	<0.001	<0.001		
ESS	0.13	−0.12	−0.05	0.04	−0.04	−0.22	0.13	0.22	0.70	
	<0.001	<0.001	0.12	0.23	0.21	<0.001	<0.001	<0.001	<0.001	
QL	0.09	−0.14	0.12	0.15	0.09	−0.36	0.63	0.59	0.30	0.25
	0.003	<0.001	<0.001	<0.001	0.002	<0.001	<0.001	<0.001	<0.001	<0.001

NrT = number of treatments; Edu = education; PI = personal income; W = working at time of diagnosis; FI = financial difficulties; RF = role functioning; SF = social functioning; ISS = instrumental social support; ESS = emotional social support; QL = quality of life.

**Table 4:** Regression coefficients for predicting global health status/QoL among cancer survivors.

		All Survivors Adjusted R^2^: 0.57					Women Adjusted R^2^: 0.59					Men Adjusted R^2^: 0.49			
		F	Df1	Df2	*p*		F	Df1	Df2	*p*		F	Df1	Df2	*p*
		34.72	19	466	<0.001		26.56	18	305	<0.001		9.50	18	143	<0.001
Predictor	β	B	SE	t	*p*	β	B	SE	t	*p*	β	B	SE	t	*p*
Intercept		54.45	7.91	6.88	<0.001 ***		50.37	9.46	5.32	<0.001 ***		63.92	15.61	4.10	<0.001 ***
Age	−0.11	−0.18	0.07	−2.54	0.01 *	−0.07	−0.12	0.09	−1.35	0.18	−0.15	−0.24	0.13	−1.86	0.06
Sex (ref. *men*)															
Women	0.03	0.68	1.65	0.41	0.68		-	-	-	-		-	-	-	-
Education (ref. *primary education*)															
Secondary school	0.23	4.72	1.95	2.42	0.02 *	0.21	4.37	2.29	1.91	0.06	0.33	6.23	4.24	1.47	0.14
Trade school	0.26	5.39	2.07	2.60	0.01 *	0.20	4.18	2.89	1.45	0.15	0.41	7.88	3.65	2.16	0.02 *
University degree	0.24	4.92	1.91	2.58	0.01 **	0.28	5.96	2.33	2.56	0.01 *	0.25	4.68	3.89	1.20	0.23
Marital status (ref. *single*)															
Widow/widower	0.36	7.44	3.92	1.90	0.06	0.41	8.65	4.47	1.94	0.053	0.16	3.05	9.20	0.33	0.74
Married or in a relationship	−0.01	−0.20	1.79	−0.11	0.91	−0.01	−0.15	2.13	−0.07	0.94	0.03	0.56	3.59	0.16	0.88
Children under 18 (*ref. no children*)	−0.04	−0.91	0.85	−1.07	0.28	−0.02	−0.36	1.09	−0.33	0.74	−0.09	−1.80	1.44	−1.25	0.21
Personal income (ref. *0–300 thousand*)															
300–500	−0.01	−0.28	1.83	−0.15	0.88	−0.02	−0.39	2.08	−0.19	0.85	−0.19	−3.67	4.63	−0.79	0.43
501–700	−0.01	−0.23	2.13	−0.11	0.91	−0.05	−1.13	2.57	−0.44	0.66	−0.16	−3.12	4.72	−0.66	0.51
701–1 million or more	−0.08	−1.73	2.38	−0.73	0.47	−0.07	−1.38	3.03	−0.45	0.65	−0.34	−6.41	4.93	−1.30	0.20
Working at diagnosis (*ref. no*)	0.08	1.70	1.76	0.96	0.34	0.09	1.90	2.16	0.88	0.38	0.16	3.08	3.44	0.90	0.37
Number of treatments (*ref. no treatment*)	0.01	0.21	0.69	0.30	0.77	−0.01	−0.14	0.80	−0.18	0.86	0.07	1.77	1.58	1.12	0.26
EORTC Role Functioning	0.26	0.19	0.03	6.22	<0.001 ***	0.23	0.17	0.04	4.66	<0.001 ***	0.35	0.24	0.06	4.15	<0.001 ***
EORTC Social Functioning	0.14	0.11	0.03	3.37	<0.001 ***	0.18	0.14	0.04	3.61	<0.001 ***	0.04	0.03	0.06	0.42	0.68
EORTC Financial Difficulties	−0.08	−0.05	0.02	−2.34	0.02 *	−0.07	−0.04	0.03	−1.74	0.08	−0.14	−0.07	0.04	−1.92	0.06
Instrumental social support	0.12	0.77	0.28	2.71	0.01 **	0.12	0.83	0.36	2.34	0.03 *	0.09	0.57	0.50	1.14	0.26
Emotional social support	−0.01	−0.21	1.01	−0.20	0.84	−0.02	−0.43	1.24	−0.35	0.79	−0.02	−0.36	1.92	−0.19	0.85
PHQ–9 symptoms of depression	−0.42	−1.75	0.17	−10.32	<0.001 ***	−0.41	−1.76	0.21	−8.51	<0.001 ***	−0.39	−1.62	0.33	−4.89	<0.001 ***

Signif. codes: *** = 0.001, ** = 0.01, * = 0.05.

**Table 5:** Regression coefficients for predicting global health status/QoL among cancer survivors—by cancer type.

		Breast Cancer Adjusted R^2^: 0.57					Prostate Cancer Adjusted R^2^: 0.48					Colorectal Cancer Adjusted R^2^: 0.54			
		F	Df1	Df2	*p*		F	Df1	Df2	*p*		F	Df1	Df2	*p*
		14.60	19	176	<0.001		6.38	17	84	<0.001		6.47	18	67	<0.001
Predictor	β	B	SE	t	*p*	β	B	SE	t	*p*	β	B	SE	t	*p*
Intercept		52.55	16.65	3.16	0.001 **		42.72	23.40	1.83	0.07		54.07	24.46	2.21	0.03 *
Age	−0.14	−0.26	0.13	−1.96	0.052	−0.03	−0.08	0.30	−0.28	0.78	0.10	0.19	0.21	0.87	0.39
Sex (ref. *men*)															
Women	0.69	13.71	10.08	1.36	0.18		-	-	-	-	0.31	5.54	3.35	1.65	0.10
Education (ref. *primary education*)															
Secondary school	0.50	9.87	2.98	3.31	0.001 **	0.44	7.42	6.72	1.10	0.27	−0.22	−3.92	4.89	−0.80	0.43
Trade school	0.46	9.15	3.59	2.54	0.012 *	0.17	2.91	5.82	0.50	0.62	−0.22	−3.94	5.34	−0.74	0.46
University degree	0.41	8.15	3.02	2.70	0.008 **	0.40	6.82	6.36	1.07	0.29	−0.08	−1.38	4.77	−0.29	0.77
Marital status (ref. *single*)															
Widow/widower	0.27	5.43	5.08	1.07	0.29	0.30	4.99	9.17	0.54	0.59	0.06	1.12	13.64	0.08	0.93
Married or in a relationship	−0.03	−0.52	2.91	−0.18	0.86	0.10	1.72	4.63	0.37	0.71	−0.19	−3.41	3.93	−0.87	0.39
Children under 18 (ref. *no children*)	−0.03	−0.58	1.43	−0.41	0.69	−0.04	−1.02	2.26	−0.45	0.65	−0.06	−1.66	2.50	−0.66	0.51
Personal income (ref. *0–300 thousand*)															
300–500	0.19	3.71	2.73	1.36	0.17	−0.50	−8.46	5.01	−1.69	0.10	0.21	3.78	5.28	0.72	0.48
501–700	0.10	2.02	3.31	0.61	0.54	−0.18	−3.03	5.22	−0.58	0.56	0.63	11.24	5.51	2.04	0.045 *
701–1 million or more	−0.04	−0.85	3.82	−0.22	0.82	−0.35	−5.96	5.09	−1.17	0.24	0.55	9.72	5.91	1.64	0.10
Working at diagnosis (*ref. no*)	−0.05	−1.08	2.93	−0.37	0.71	0.12	2.09	3.53	0.59	0.56	0.34	5.98	4.18	1.43	0.16
Number of treatments (*ref. no treatment*)	−0.10	−2.30	1.24	−1.85	0.07	0.02	0.48	1.85	0.26	0.80	−0.05	1.14	2.04	−0.56	0.58
EORTC Role Functioning	0.27	0.21	0.05	4.38	<0.001 ***	0.47	0.40	0.09	4.50	<0.001 ***	0.47	0.29	0.07	4.36	<0.001 ***
EORTC Social Functioning	0.12	0.09	0.05	1.80	0.07	0.11	0.09	0.10	0.94	0.35	0.04	0.03	0.08	0.33	0.75
EORTC Financial Difficulties	−0.10	−0.06	0.03	−1.88	0.06	0.04	0.02	0.05	0.41	0.69	−0.11	−0.06	0.05	−1.12	0.27
Instrumental social support	0.10	0.58	0.46	1.25	0.21		-	-	-	-		-	-	-	-
Emotional social support	0.001	0.03	1.59	0.16	0.99	−0.01	−0.30	1.93	−0.15	0.88	−0.12	−3.59	2.45	−1.47	0.15
PHQ-9 symptoms of depression	−0.41	−1.74	0.28	−6.32	<0.001 ***	−0.37	−2.21	0.60	−3.71	<0.001 ***	−0.23	−1.12	0.55	−2.03	0.046 *

Signif. codes: *** = 0.001, ** = 0.01, * = 0.05.

## Data Availability

The datasets presented in this article are not readily available because of personal protection laws. Requests to access the datasets should be directed to the corresponding author.
